# Strengthening public health surveillance and response using the health systems strengthening agenda in developing countries

**DOI:** 10.1186/1471-2458-10-S1-S5

**Published:** 2010-12-03

**Authors:** Peter Nsubuga, Okey Nwanyanwu, John N Nkengasong, David Mukanga, Murray Trostle

**Affiliations:** 1 Field Epidemiology and Laboratory Training Program and Systems (Africa) Branch, Division of Public Health Systems and Workforce Development, Center for Global Health, CDC, Atlanta GA, USA; 2 Global AIDS Program, Center for Global Health, CDC, Atlanta GA, USA; 3 International Laboratory Branch Global AIDS Program, Center for Global Health, CDC, Atlanta GA, USA; 4 African Field Epidemiology Network, Kampala, Uganda; 5 Bureau for Global Health, U.S. Agency for International Development, Washington DC, USA

## Abstract

There is increased interest in strengthening health systems for developing countries. However, at present, there is common uncertainty about how to accomplish this task. Specifically, several nations are faced with an immense challenge of revamping an entire system. To accomplish this, it is essential to first identify the components of the system that require modification. The World Health Organization (WHO) has proposed health system building blocks, which are now widely recognized as essential components of health systems strengthening.

With increased travel and urbanization, the threat of emerging diseases of pandemic potential is increasing alongside endemic diseases such as human immunodeficiency virus (HIV), tuberculosis (TB), malaria, and hepatitis virus infections. At the same time, the epidemiologic patterns are shifting, giving rise to a concurrent increase in disease burden due to non-communicable diseases. These diseases can be addressed by public health surveillance and response systems that are operated by competent public health workers in core public health positions at national and sub-national levels with a focus on disease prevention.

We describe two ways that health ministries in developing countries could leverage President Obama’s Global Health Initiative (GHI) to build public health surveillance and response systems using proven models for public health systems strengthening and to create the public health workforce to operate those systems. We also offer suggestions for how health ministries could strengthen public health systems within the broad health systems strengthening agenda. Existing programs (e.g., the Global Vaccine Alliance [GAVI] and the Global Fund Against Tuberculosis, AIDS, and Malaria [GFTAM]) can also adapt their current health systems strengthening programs to build sustainable public health systems.

## Background and context

Despite increased interest in strengthening health systems for developing countries, the current reality is that the health systems in most developing countries fall short of the requirements to implement the goals suggested by the World Health Organization (WHO) International Health Regulations (IHR[2005]). One of the greatest obstacles that countries face is a clear understanding of the steps that are needed to strengthen a health system. To address this issue, the WHO has proposed health system building blocks which are now widely recognized as essential components of health systems strengthening [1]. These building blocks include service delivery, financing, governance, the health workforce, information systems, and supply management systems. The proposed building blocks address the full range of services required for an effective public health system and include both treatment-based and preventive strategies. The WHO has also recently launched an intervention to address the alarming shortage of health workers in developing countries, which includes curative and preventive public health staff [2].

The threats posed by emerging pandemic threats intensify the need to develop worldwide capacity for public health surveillance and response. With increased travel and urbanization, the threat of emerging diseases of pandemic potential is increasing. Against a backdrop of endemic diseases such as human immunodeficiency virus (HIV), tuberculosis (TB), malaria, and hepatitis virus infections, epidemiologic patterns are shifting, giving rise to significant increases in disease burden due to of non-communicable diseases. Good international public health surveillance and response, which is the basis of the revised IHR, cannot exist sustainably without good domestic surveillance and response. Both IHR(2005) and domestic public health surveillance and response require public health systems that are operated by competent public health workers in core public health positions at national and sub-national levels with a focus on disease prevention. However, when officials seek to address these public health issues, they are faced with several interrelated problems including human resource capacity, disease surveillance and reporting capacity, and response capacity. Clearly, an integrated and sustainable approach that enables the development of public health worker capacity will be critical to achieving public health surveillance and response systems that have a sustainable and adaptable capacity to address evolving public health needs.

An example of the urgent need to develop public health surveillance and response systems in developing countries is the 2009 influenza A H1N1, the first pandemic of the 21^st^ century, which had a significantly greater impact among individuals with underlying diseases in the northern hemisphere [3], and had more severe effects in populations with large HIV and TB burdens in the southern hemisphere [4]. In what appears to be a reversal of the usual picture that is observed with emerging infectious diseases, influenza A H1N1 was exported from developed nations with adequate public health surveillance and response systems to developing nations with inadequate public health surveillance and response systems [5], and could have had devastating effects.

We describe two ways that health ministries in developing countries could leverage U.S. President Obama’s Global Health Initiative (GHI), to build public health systems using proven models for public health systems strengthening and create the public health workforce to operate those systems. Specifically, we discuss the successful Field Epidemiology Training Program (FETP) and the Field Epidemiology and Laboratory Training Program (FELTP), and efforts to establish integrated disease surveillance and response within developing countries. We also offer suggestions for how health ministries could strengthen public health systems within the broad health systems strengthening agenda. Existing programs (e.g., the Global Vaccine Alliance [GAVI] and the Global Fund Against Tuberculosis, AIDS and Malaria [GFTAM]) can also adapt their current health systems strengthening programs to build sustainable public health systems.

## The role of Field Epidemiology and Laboratory Training Programs in public health systems strengthening

GHI will provide an opportunity for major investments in sustainable health systems strengthening and public health systems strengthening in developing countries. With an estimated USD 63 billion investment in global health, GHI will be the principal engine for global health development for the foreseeable future. The President’s Emergency Plan for AIDS Relief (PEPFAR) reauthorization, which is part of GHI, includes the goal of pre-service training for 140,000 new health care workers within five years in the recipient countries [8]. We believe that these new health workers will need to respond by providing both treatment and preventive service, and that the number of public health workers needed to make a significant change in the operation of public health systems is substantially less than number of health care workers needed for curative health care services. While the focus of public health system strengthening has previously been aimed at treatment of disease, the advent of emerging pandemics necessitates incorporation of other specialties including epidemio-logic, laboratory and management expertise. In our experience, a successful way to strengthen public health surveillance and response systems is through Field Epidemiology and Laboratory Training Programs (FETPs and FELTPs) or allied programs (e.g., Public Health Schools Without Walls) [9] and through competency-based short courses, mainly those in basic field epidemiology for frontline surveillance and response staff [10].

FETPs /FELTPs provide a critical component of the public health workforce that is needed to operate public health surveillance and response systems to implement the IHR(2005) [11]. FETPs/FELTPs are modeled on the successful Epidemic Intelligence Service (EIS) training that has been offered by the U.S. Centers for Disease Control and Prevention (CDC) since the 1950s. EIS has been responsible for developing U.S. public health surveillance and response systems at the federal and state level. FETPs/FELTPs are often started with donor funding (e.g., the U.S. Agency for International Development [USAID], the World Bank, and lately PEPFAR) and CDC technical assistance, and are designed to transition to host government funding. FETPs/FELTPs train public health personnel using a two-year long competency-based residency approach where trainees provide public health service to the ministry of health during their training. Many FETP/FELTP programs offer a masters degree or the equivalent at the end of the two-year postgraduate training. Graduates of the programs are able to operate national and sub-national public health surveillance and response systems, with growing responsibility as they gain experience. Increasingly FETPs/FELTPs are designed with a goal of contributing to the following critical outcomes within five to ten years after startup in the host country:

a) functional and robust public health surveillance systems, often beginning with notifiable disease surveillance systems and moving on to add non-communicable disease surveillance systems;

b) prompt and effective response to public health emergencies, including disease outbreaks and other acute public health events;

c) a culture of evidence-based decision making in public health whereby programmatic decisions are made on scientifically sound data;

d) a strengthened, motivated public health workforce comprising public health leaders (i.e., graduates of the two-year FETP/FELTP course) and frontline public health implementers (i.e., graduates of the in the short courses that are associated with the program); and

e) reduction in morbidity and mortality from priority diseases in the host country.

Although no precise studies have been done to establish a target for the public health workforce, for a basic multi-disease public health surveillance and response system to be operational in a developing country, certain core public health positions and structures are critically needed at the national and sub-national level. At the sub-national level, a province, or region, comprised of several districts is commonly the point of primary public health program implementation. We believe that a province or region should have at least three FETP/FELTP graduates to operate basic multi-disease public health surveillance and response systems: one to lead communicable and non-communicable disease surveillance; one to lead communicable and non-communicable disease control; and one to lead the public health laboratory network for the province. These graduates should be working together within a provincial disease surveillance and disease control unit reporting to the provincial medical director and with a complement of district-based frontline staff that are trained in basic field epidemiology and public health laboratory practice as they jointly operate public health systems within the province. Larger provinces may need to have more graduates to address specific diseases and graduates may even be deployed at the district level in smaller countries.

At the national level, a team of FETP/FELTP graduates working in a national multi-disease surveillance, disease control, and public health laboratory unit or department should be linked to the provincial disease surveillance, disease control, and public health laboratory units to form a robust national multi-disease surveillance and response network. More FETP/FELTP graduates may be needed for specific programmatic needs at the national level (e.g., to operate the HIV, TB or malaria programs). Some health ministries may need to train a cadre of public health managers and logisticians for the public health systems to operate efficiently. The multi-disease surveillance, disease control, and laboratory network positions at national and sub-national level would then form the core public health positions that are necessary for essential public health actions to occur within the country. The national and provincial multi-disease surveillance and disease control departments and units would form the network through which these essential public health functions would be conducted.

We believe that a country would have an adequate coverage of public health workers trained in the FETP or FELTP if there are three to five graduates per million inhabitants in the country, to reach adequate coverage an FETP or FELTP would need to be operating for more than 5-10 years.

FETPs/FELTPs have developed international and regional networks (e.g., Training in Public Health Interventions Network (TEPHINET, http://www.tephinet.org) [12], and the African Field Epidemiology Network (AFENET, http://www.afenet.net) [13]), that are playing a major role in networking trainees and graduates for effective public health surveillance and response systems. AFENET, for example, is supporting graduates to implement various public health surveillance and response activities in the health ministries of member countries, and TEPHINET and AFENET trainees and graduates have participated in international outbreak investigation and management teams under the auspices of the WHO.

Many FETPs/FELTPs are currently operated by their host country nationals and have transitioned from direct donor funding. Examples of programs that have transitioned from donor funding in Africa include those in Zimbabwe and Uganda. Most programs have a steering committee that is led by the ministry of health and includes all important stakeholders (e.g., the host country university, donors, the WHO, etc.) The steering committee shepherds that vision of the program which includes career paths for the graduates, a plan for sustainability, and a plan for transitioning from donor funding. Many programs are led by graduates of the initial cohorts of trainees, including those in Brazil, Thailand, and Kenya.

## Building effective and adaptable frameworks for integrated disease surveillance and response in resource-constrained countries

Several programs aim to improve public health surveillance and response in developing countries by addressing specific disease control needs (e.g., vaccine preventable disease surveillance and response or HIV/AIDS), often in line with donor perspectives. There are also crosscutting initiatives aiming to improve the general public health surveillance and response system in a multi-disease manner. The WHO’s Integrated Disease Surveillance and Response Strategy (IDSR), which is being implemented in all 46 Member States of the WHO’s African Regional Office and in the Integrated Disease Surveillance and Response Project in India, are examples of general crosscutting public health and response improvement programs that have originated in developing countries. With sustained support from USAID and other donors from 1998 to date, IDSR has been successful because in addition to being a threshold-based surveillance strategy that focuses on public health response at the district or equivalent level, its implementation has gone through a process which allows all stakeholders to achieve a shared vision of what good multi-disease public health surveillance and response can look like in their country. Th]e IDSR process starts with a baseline in-depth assessment and analysis of gaps, and then development of prioritized plans of action, which are implemented by the various stakeholders in a coordinated manner and are monitored, evaluated, and improved [14,15]. Currently, IDSR is the platform on which IHR implementation in Africa will be built and it is moving to address non-communicable diseases.

PEPFAR has supported initiatives to strengthen public health laboratory systems to address multiple diseases in resource-constrained settings by leveraging and integrating all the support for laboratory services, after the development of national laboratory strategic plans [16,17]. The strategic plans include consideration for policy, legal, and regulatory frameworks, the administrative and technical management structure of the laboratories, human resources and retention strategies, laboratory quality management systems, monitoring and evaluation systems, procurement and maintenance of equipment, and laboratory infrastructure enhancement. Several countries have developed or are in the process of developing their laboratory plans, and others, such as Ethiopia, have implemented and evaluated their plans [17][20].

We propose that health ministries in developing countries adopt the following suggestions as they grapple with the challenges of strengthening public health systems within the broader challenge of health systems strengthening:

1) Devote at least as much attention to public health as is given to treatment-focused health efforts in all aspects of health systems strengthening in order to lay adequate emphasis on public health systems strengthening within broader health systems strengthening.

2) Leverage GHI and other multilateral and bilateral funding to ensure that some of those resources are used to develop sustainable public health systems, with a focus on developing and retaining the public health workforce in core public health positions at national and sub-national level to operate the strengthened systems.

3) Adapt existing public health system frameworks (e.g., IDSR), including the processes that lead to a shared vision, a common strategic plan, and a common set of indicators to other priority public health conditions (e.g., maternal and child health, non-communicable diseases, and environmental hazards).

4) Support the ongoing process of strengthening public health laboratory services using a multi-disease approach through one national strategic plan and coordinated and leveraged investments.

5) Implement training programs for public health leaders and frontline public health workers within the country with a focus on critical outcomes that are measured and improved upon incrementally.

6) Leverage existing funding mechanisms like GAVI and GFATM to develop sustainable public health systems which are operated by competently training public health workers.

Using approaches such as these will ensure that the current interest in health systems strengthening is translated into sustainable public health strengthening along with curative health system strengthening which is the main focus of current efforts. Preventive or public health system strengthening will be critical to address the myriad of health challenges that are faced by developing countries including IHR and the Millennium Development Goals, particularly those that address public health issues.

## Summary and conclusion

Developing countries need a public health workforce to operate public health surveillance and response systems, good domestic public health surveillance and response is necessary for implementation of IHR(2005). FETPs and FELTPs provide a proven strategy to develop a locally-trained public health workforce for public health surveillance and response, and IDSR provides a strategy that can be used to develop a basic multi-disease surveillance and response system that can be operated by FETP and FELTP graduates. We strongly suggest that development partners should support developing countries to try both these strategies as one way to ensure implementation of IHR as well as domestic surveillance and response priorities.

## Abbreviations

AFENET: African Field Epidemiology Network; FELTP: Field Epidemiology and Laboratory Training Program; FETP: Field Epidemiology Training Program; GAVI: Global Vaccine Alliance; GFTAM: Global Fund Against Tuberculosis, AIDS and Malaria; GHI: Global Health Initiative; HIV: Human immunodefciency virus; IDSR: Integrated Disease Surveillance and Response Strategy; IHR: International Health Regulations; PEPFAR: President’s Emergency Plan for AIDS Relief; TB: Tuberculosis; TEPHINET: Training in Public Health Interventions Network; USAID: United States Agency for International Development; WHO: World Health Organization.

## Competing interests

No competing interests

## Authors’ contributions

All the authors participated in conceiving the idea for the paper and helped with writing sections of the paper. All authors reviewed and agreed with the contents of the paper. Peter Nsubuga is the corresponding author.
